# Pre-operative to post-operative serum carcinoembryonic antigen ratio is a prognostic indicator in colorectal cancer

**DOI:** 10.18632/oncotarget.17931

**Published:** 2017-05-17

**Authors:** Zhenqiang Sun, Fuqi Wang, Quanbo Zhou, Shuaixi Yang, Xiantao Sun, Guixian Wang, Zhen Li, Zhiyong Zhang, Junmin Song, Jinbo Liu, Weitang Yuan

**Affiliations:** ^1^ Department of Anorectal Surgery, First Affiliated Hospital of Zhengzhou University, Zhengzhou 450052, China

**Keywords:** colorectal cancer, CEA, tumor marker, prognosis

## Abstract

We explored the prognostic significance of the pre-operative-to-post-operative serum carcinoembryonic antigen (pre-post-CEA) ratio in colorectal cancer (CRC). We detected pre- and post-operative CEA levels in 2035 CRC patients surgically treated at First Affiliated Hospital of Zhengzhou University between June 2001 and June 2011. Univariate analysis revealed the pre-post-CEA ratio is associated with distant metastasis and degree of tumor differentiation (both *P* < 0.05). Multivariate analysis showed that the pre-post-CEA ratio is associated with lymphatic and distant metastasis, tumor-node-metastasis (TNM) stage and degree of tumor differentiation (all *P* < 0.01). The pre-CEA levels, pre-post-CEA ratios, distant metastasis, TNM stage and degree of tumor differentiation were all associated with 5-yr overall survival (all *P* < 0.05) based on multivariate analysis. Consequently, pre-CEA levels, pre-post-CEA ratios, distant metastasis and TNM stage are independent risk factors for CRC. We have thus demonstrated that the pre-post-CEA ratio is a prognostic indicator for CRC patients.

## INTRODUCTION

Colorectal cancer (CRC) is the third most commonly diagnosed cancer and the fourth leading cause of cancer death worldwide with steadily increasing mortality rates [1, 2]. In recent years, lifestyle changes including high-fat diet, lack of exercise and mental stress has resulted in a rise in CRC cases [3]. Radical surgery still remains the best therapeutic option for CRC patients [4]. However, high recurrence and metastasis rates have resulted in poor overall survival of CRC patients [5, 6]. Therefore, identification of novel prognostic risk factors is necessary for improving survival rates.

In recent years, tumor biomarkers have been widely used in clinical diagnosis, post-operative monitoring of tumor recurrence, prognosis and curative therapy of CRC patients [7]. The serum carcinoembryonic antigen (CEA) is one of the tumor biomarkers used for predicting recurrence, prognosis and therapeutic efficacy in CRC patients [8, 9]. The potential clinical use of changes in pre-operative and post-operative CEA levels (pre-post-CEA) has been recognized in few cancer studies [10, 11]. Increased serum CEA levels were observed after radical surgery in some patients [12, 13]. The significance of pre- and post-operative CEA levels is controversial. Hotta *et al*. reported that the pre-post-CEA ratio is prognostic predictor after surgery for stage III rectal cancer patients [10]. However, another study reported that post-operative serum CEA (post-CEA) levels were more definitive as prognostic prediction than pre-post-CEA ratios in non-small cell lung cancer patients. Therefore, in this study, we explored the prognostic relevance of pre-post-CEA ratios in predicting the survival time of CRC patients after radical surgery.

## RESULTS

### Selection strategy of research objects

**Figure 1:**
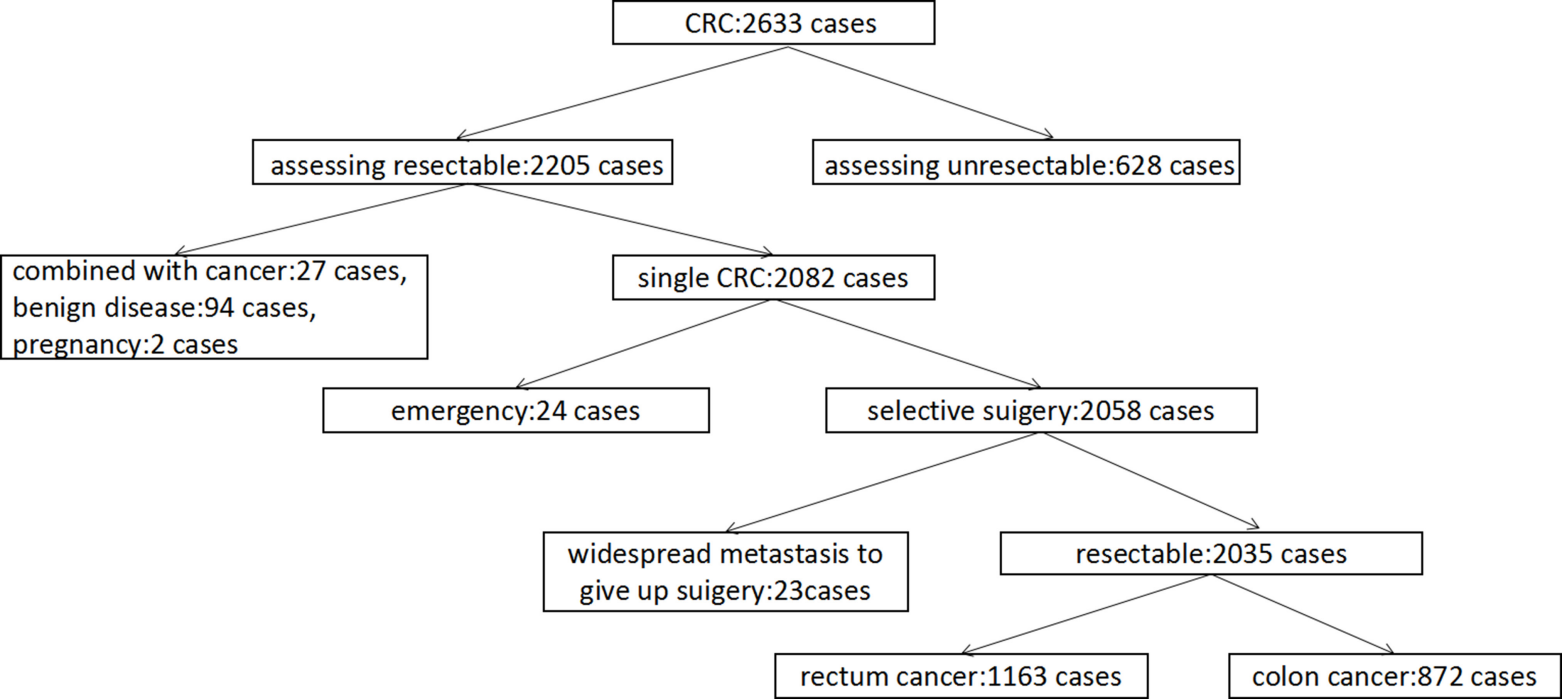
Scheme of selecting research subjects After screening 2833 CRC cases for eliminating criteria including unresectable tumors, combined with other cancers, benign disease, pregnancy, emergency and widespread metastasis, 2035 CRC cancer patients were chosen for the study.

After screening 2833 CRC patients, we excluded the following: (1) unresectable (628 cases); (2) combined with other cancers (27 cases); (3) benign disease (94 cases); (4) pregnancy (2 cases); (5) emergency (24 cases) and (6 cases) untreatable due to widespread metastasis (23 cases). Finally, we enrolled 2035 patients including 1138 males and 897 females; of these, 872 had colon cancer and 1163 had rectal cancer (Figure 1).

### Univariate analysis of correlation between pre-CEA levels and pre-post-CEA ratios with clinicopathological parameters of CRC patients

The patients were divided into low and high expression groups based on pre-CEA levels and pre-post-CEA ratios based on their mean values. Univariate analysis showed that pre-CEA levels were associated with smoking, tumor size, lymphatic and distant metastases and TNM stage (*P* < 0.05 for all parameters; Table 1). The pre-post-CEA ratios were associated with distant metastasis and degree of tumor differentiation (both *P* < 0.05; Table 1). Pre-CEA levels were not associated with gender, age, family history, tissue type, degree of tumor differentiation and tumor location. Meanwhile, pre-post-CEA ratios were not associated with gender, age, family history, smoking, tumor size, lymphatic metastasis, TNM stage, tissue type and tumor location (all *P* > 0.05).

**Table 1:** Univariate analysis of relationship between pre-CEA levels and pre-post-CEA ratios with clinicopathological parameters of CRC patients

Clinicopathologic characteristics	*n*	Pre-CEA expression		X^2^	*P* value	Pre-post-CEA ratio		X^2^	*P* value
		Low	High			Low	High		
**Gender**				0.85	0.357			1.381	0.240
Male	1138	467	671			303	863		
Female	897	350	547			206	663		
**Age (Years)**				5.562	0.180			0.005	0.943
≥ 60	1091	412	679			250	737		
< 60	944	405	539			264	784		
**Family history**				3.504	0.610			0.839	0.36
Yes	173	81	92			68	179		
No	1862	736	1126			444	1344		
**Smoking**				4.016	0.045			0.508	0.476
Yes	426	153	273			122	337		
No	1609	664	945			393	1183		
**Tumor size (cm)**				4.509	0.034			0.624	0.429
≥ 5 cm	1288	378	622			273	832		
< 5 cm	817	439	596			244	686		
**Lymphatic metastasis**				5.554	0.018			0.200	0.655
Yes	964	361	603			238	687		
No	1071	456	615			276	834		
**Distant metastasis**				179.119	< 0.001			40.936	<0.001
Yes	615	111	504			140	224		
No	1420	706	714			374	1297		
**TNM stage**				90.384	< 0.001			1.220	0.269
I/II	641	355	286			243	760		
III/IV	1394	462	932			272	760		
**Tissue type**				0.32	0.858			0.538	0.463
Adenocarcinoma	1551	621	930			424	1279		
Non-adenocarcinoma	484	196	288			89	243		
**General tumor type**				0.093	0.76			0.516	0.473
Ulcerative	1174	468	706			289	885		
Non-ulcerative	861	349	512			224	637		
**Differentiation degree**				2.344	0.120			40.037	< 0.001
High/Median	1291	502	789			326	1187		
Low/undifferentiated	744	315	429			188	334		
**Tumor location**				1.43	0.232			2.389	0.122
Colon	872	337	535			234	595		
Rectum	1163	480	683			379	827		

### Multivariate analysis of the correlation between pre-CEA levels and pre-post-CEA ratios with clinicopathological parameters of CRC patients

Multivariate analysis showed that pre-CEA levels were associated with family history, smoking, tumor size, distant metastasis and TNM stages (all *P* < 0.05; Table 2). However, they were not associated with other cancer in combination, lymphatic metastasis, tissue type, general tumor type, degree of tumor differentiation and tumor location (all *P* > 0.05). Meanwhile, pre-post-CEA ratios were associated with lymphatic and distant metastases, TNM stage and degree of tumor differentiation (all *P* < 0.01; Table 2). But, they were not associated with family history, smoking, other cancer in combination, tumor size, tissue type, general tumor type and tumor location (all *P* > 0.05).

**Table 2:** Multivariate analysis of relationship between pre-operative CEA and pre- to post-operative CEA with clinicopathological parameters of CRC patients

Clinicopathologic characteristics	Pre-operative CEA expression				Pre- to post-operative CEA ratio			
	HR	*P*	95% CI		HR	*P* value	95% CI	
			Lower	Upper			Lower	Upper
**Tumor family history** Yes vs. No	4.576	0.032	0.494	0.970	0.476	0.490	0.715	2.014
**Smoking** Yes vs. No	5.072	0.024	1.036	1.664	0.108	0.742	0.712	1.609
**Tumor size (cm)** ≥5cm vs. <5cm	9.812	0.002	1.124	1.662	1.093	0.296	0.575	1.183
**Lymphatic metastasis** Yes vs. No	0.363	0.547	0.598	1.313	20.017	0.000	2.330	8.708
**Distant metastasis** Yes vs. No	63.108	0.000	2.423	4.326	28.176	0.000	2.284	6.006
TNM stages I/II vs. III/IV	9.087	0.003	0.331	0.791	28.445	0.000	3.320	13.384
**Tissue type** Adenocarcinoma vs.\ Non-adenocarcinoma	0.099	0.753	0.789	1.388	0.051	0.821	0.594	1.512
**Tumor general type** Ulcerative vs. Non-ulcerative	0.684	0.408	0.877	1.383	3.792	0.051	0.498	1.002
**Differentiation degree** High/Median vs. Low/undifferentiated	1.077	0.299	0.904	1.388	10.381	0.001	0.353	0.776
**Tumor location** Colon vs. Rectum	0.010	0.919	0.811	1.208	1.378	0.241	0.869	1.754

### Univariate analysis of the correlation between pre-CEA levels and pre-post-CEA ratios with 5-year OS

As shown in Table 3, univariate analysis demonstrated that pre-CEA levels, pre-post-CEA ratios, tumor size, TNM stage, lymphatic metastasis, distant metastasis and degree of tumor differentiation were associated with 5-year overall survival (OS, all *P* < 0.05). Factors like age, gender, family history, smoking, tissue type and tumor location were not related with 5-year OS (all *P* > 0.05).

**Table 3:** Univariate analyses of correlation between clinicopathological parameters and 5-year OS of CRC patients

Clinicopathologic characteristics	*n*	5-year OS	X^2^	*P* value
**Gender**			0.384	0.535
Male	1138	72.2%		
Female	897	66.7%		
**Age (Years)**			0.046	0.831
≥ 60	1091	69.5%		
< 60	944	69.9%		
**Family history**			0.000	0.997
Yes	173	68.3%		
No	1862	69.7%		
**Smoking**			10282	0.258
Yes	426	75.4%		
No	1609	67.8%		
**Pre-CEA levels**			13.849	< 0.001
high	1218	60%		
low	817	79.1%		
**Pre-post-CEA ratio**			14.513	< 0.001
Low	578	62.8%		
High	195	72%		
**Tumor size (cm)**			5.526	0.019
≥ 5 cm	945	60.7%		
< 5 cm	990	76.1%		
**Lymphatic metastasis**			6.353	0.012
Yes	1024	64.2%		
No	1011	72%		
**Distant metastasis**			122.546	< 0.001
Yes	615	27.4%		
No	1420	78.2%		
**TNM stage**			4.781	0.029
I/II	641	71.6%		
III/IV	1394	65.2%		
**Tissue type**			0.858	0.354
Adenocarcinoma	1551	69.5%		
Non-adenocarcinoma	484	70.1%		
**Tumor general type**				
Ulcerative	1174	67.4%	0.976	0.269
Non-ulcerative	861	71.2%		
**Differentiation degree**			6.238	0.013
High/Median	1291	70.4%		
Low/undifferentiated	744	64.5%		
**Tumor location**			0.026	0.872
Colon	872	71.8%		
Rectum	1163	68.3%		

### Multivariate analysis of the correlation between pre-CEA levels and pre-post-CEA ratios with 5-year OS

Multivariate survival analysis showed that pre-operative CEA, pre- to post- operative CEA ratio, distant metastasis, TNM stage and degree of tumor differentiation were associated with 5-year OS (all *P* < 0.05, Table 4). Meanwhile, gender, age, family history, smoking, tumor size, lymphatic metastasis, tissue type, general tumor type and tumor location were not associated with 5-year OS (all *P* > 0.05, Table 4). Therefore, our analysis demonstrated that pre-CEA levels, pre-post-CEA ratios, distant metastasis, TNM stage and differentiation degree were independent risk factors for 5-year OS of CRC patients.

**Table 4:** COX regression survival analyses of clinicopathological parameters of CRC patients

Clinicopathological characteristics	RR	*P* value	95% confidence interval	
			Lower	Upper
**Gender** Male vs. Female	0.591	0.442	0.786	1.738
**Age (Years)** ≥ 60 vs. < 60	2.536	0.111	0.511	1.072
**Tumor family history** Yes vs. No	0.043	0.835	0.618	1.815
**Smoking** Yes vs. No	0.046	0.831	0.636	1.758
**Pre-CEA** ≥ 5 vs. < 5	7.897	0.005	0.351	0.830
**Pre-post-CEA** Low vs. High	5.434	0.020	0.390	0.922
**Tumor size (cm)** ≥ 5 cm vs. < 5 cm	2.581	0.108	0.485	1.074
**Lymphatic metastasis** Yes vs. no	0.093	0.761	0.512	1.631
**Distant metastasis** Yes vs. no	28.112	0.000	0.177	0.451
**TNM stage** I/II vs. III/IV	4.420	0.036	1.054	4.540
**Tissue type** Adenocarcinoma vs. Non-adenocarcinoma	0.568	0.451	0.471	1.398
**Tumor general type** Ulcerative vs. Non-ulcerative	1.640	0.200	0.884	1.801
**Differentiation degree** High/Median vs. Low/undifferentiated	10.181	0.001	1.272	2.741
**Tumor location** Colon vs. Rectum	0.003	0.959	0.697	1.463

### Kaplan-Meier analysis of association of overall survival with pre-CEA levels and pre-post-CEA ratios

**Figure 2:**
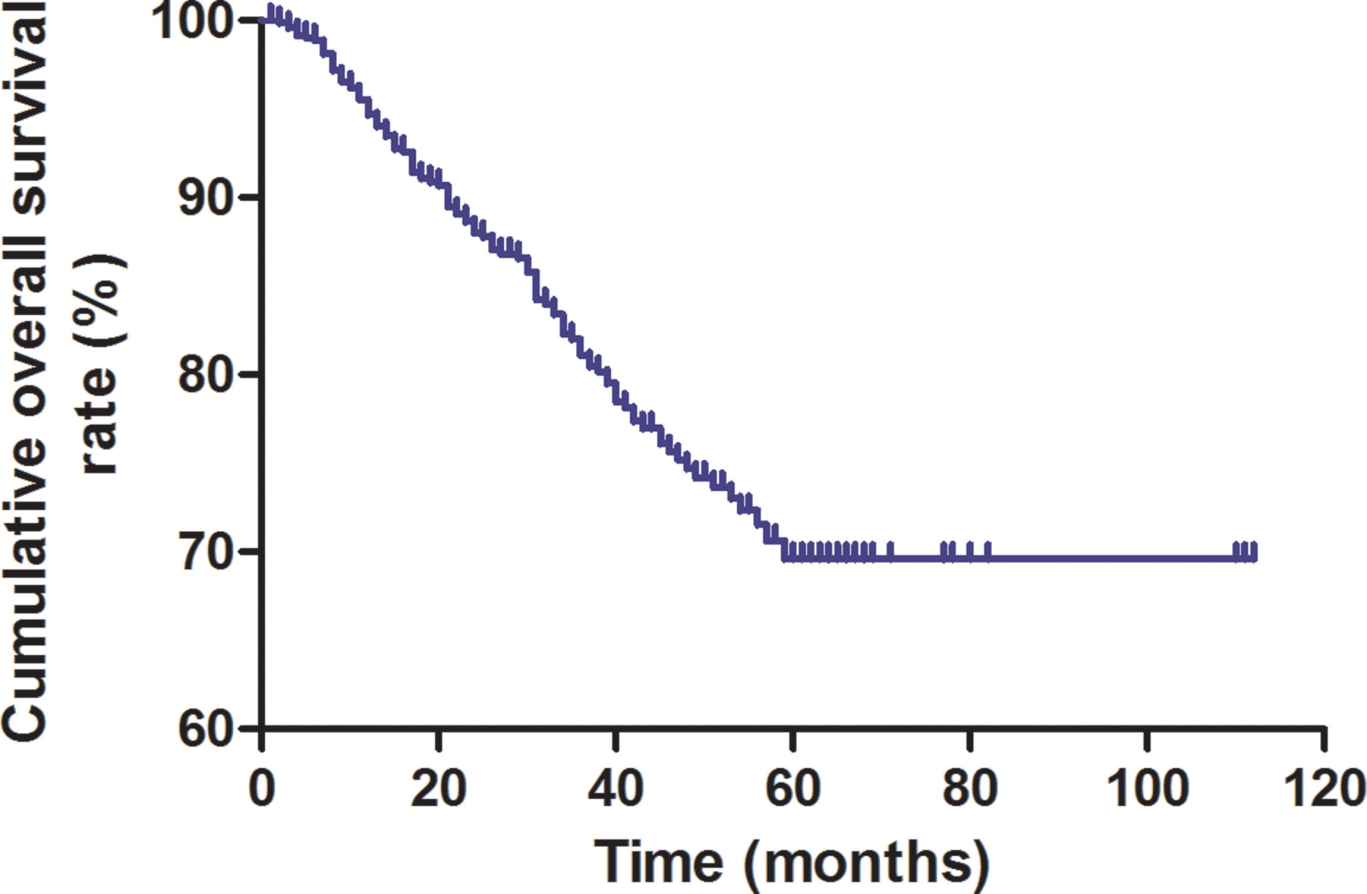
Kaplan-Meier survival curve of enrolled CRC patients The Kaplan-Meier survival plot shows decreasing overall survival with post-operative time (months).

**Figure 3:**
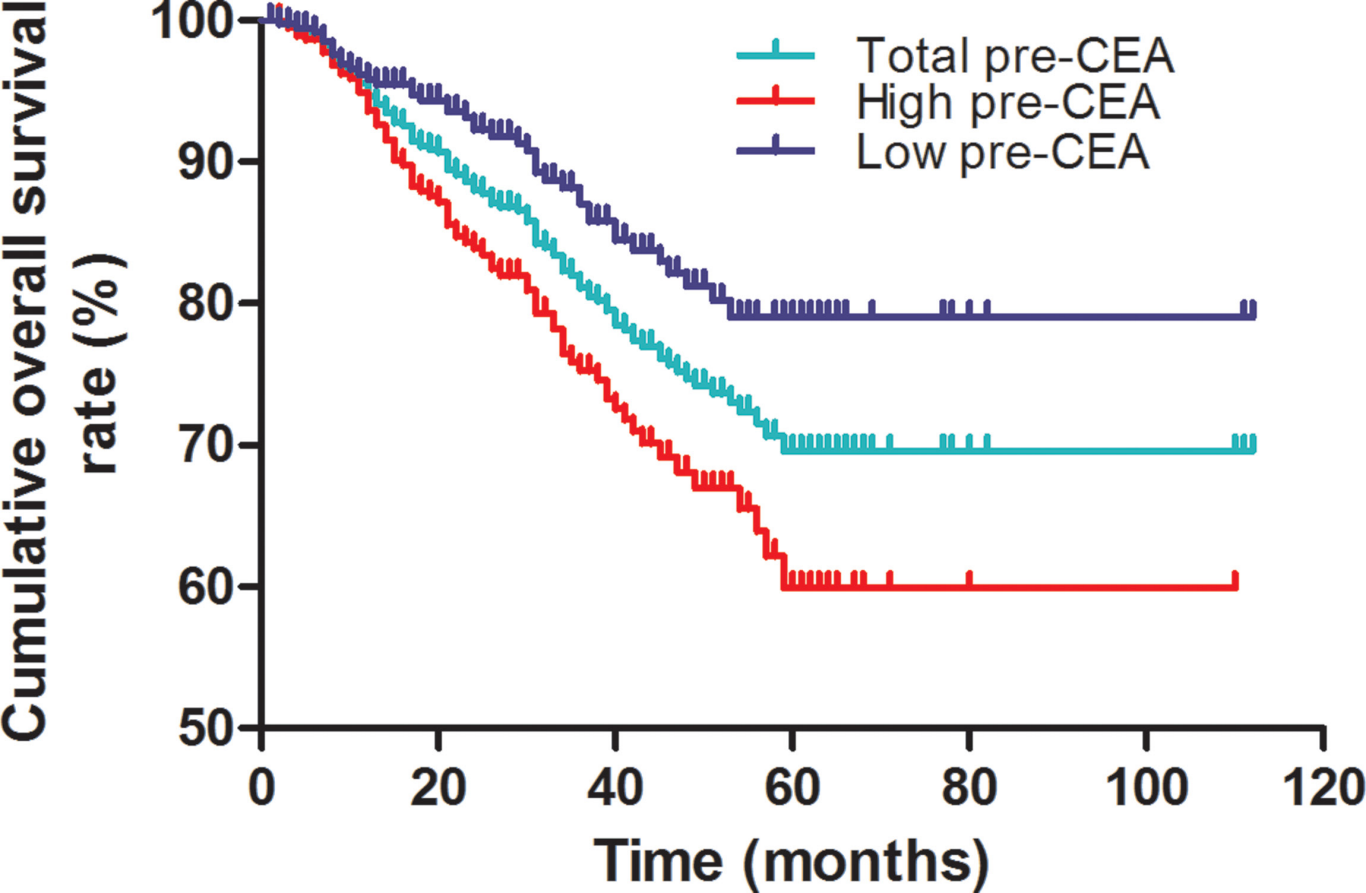
Kaplan-Meier survival curves of CRC patients based on pre-operative CEA levels Overall survival of CRC patients with high (red) and low (blue) pre-operative CEA levels compared to total (green) CRC patients based on Kaplan-Meier curves and log-rank test. As shown, patients with low pre-CEA levels survived longer than those with high pre-CEA levels (log-rank test, *P* < 0.001).

**Figure 4:**
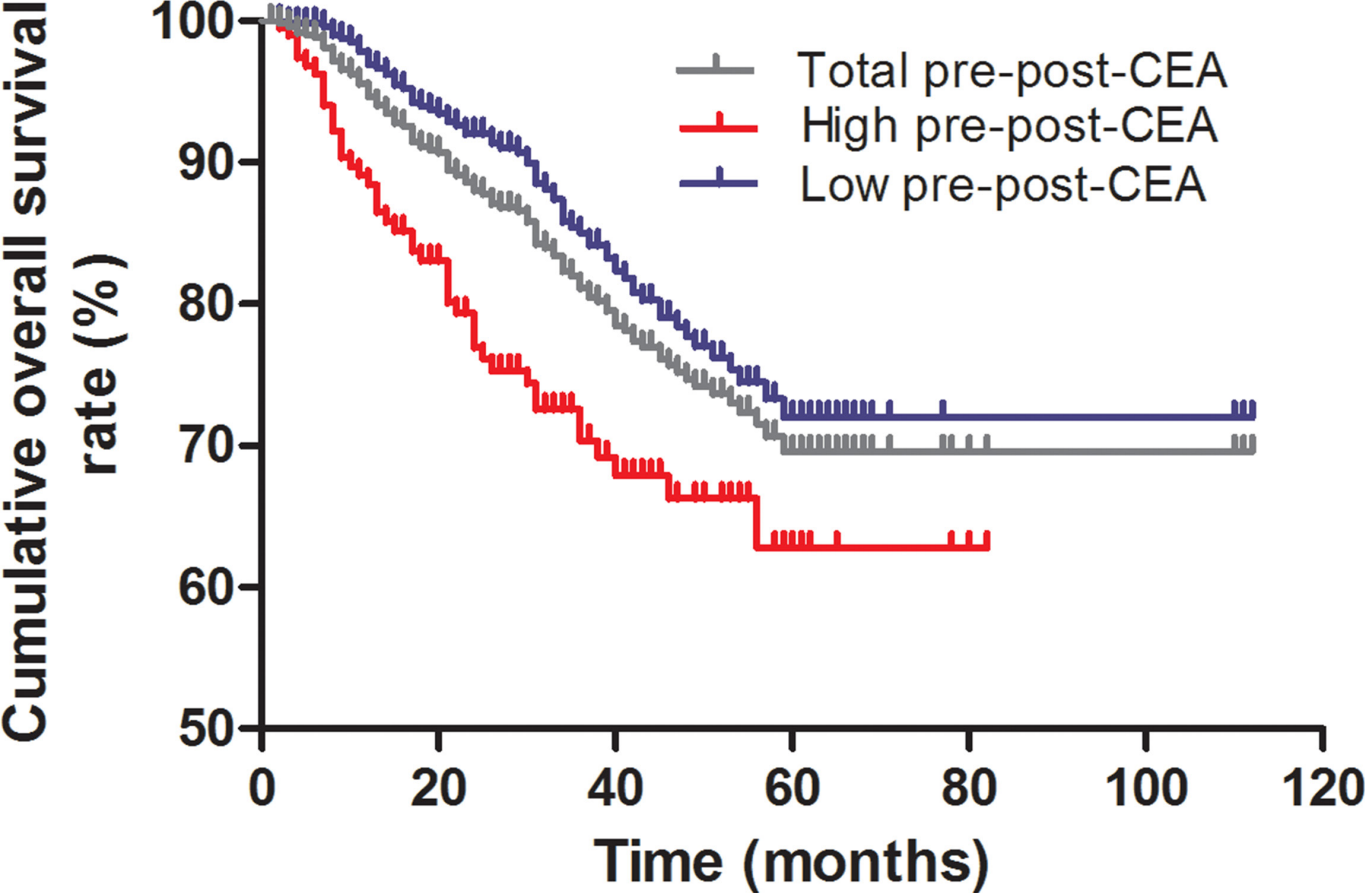
Kaplan-Meier survival curves of CRC patients based on pre-post-CEA ratios Overall survival of CRC patients with low (blue) and high (red) pre-post-CEA ratios compared to total (gray) number of CRC patients based Kaplan-Meier curves and log rank test. As shown, patients with low pre-post-CEA ratios survived longer than those with high pre-post-CEA ratios (log-rank test, *P* < 0.001).

**Figure 5:**
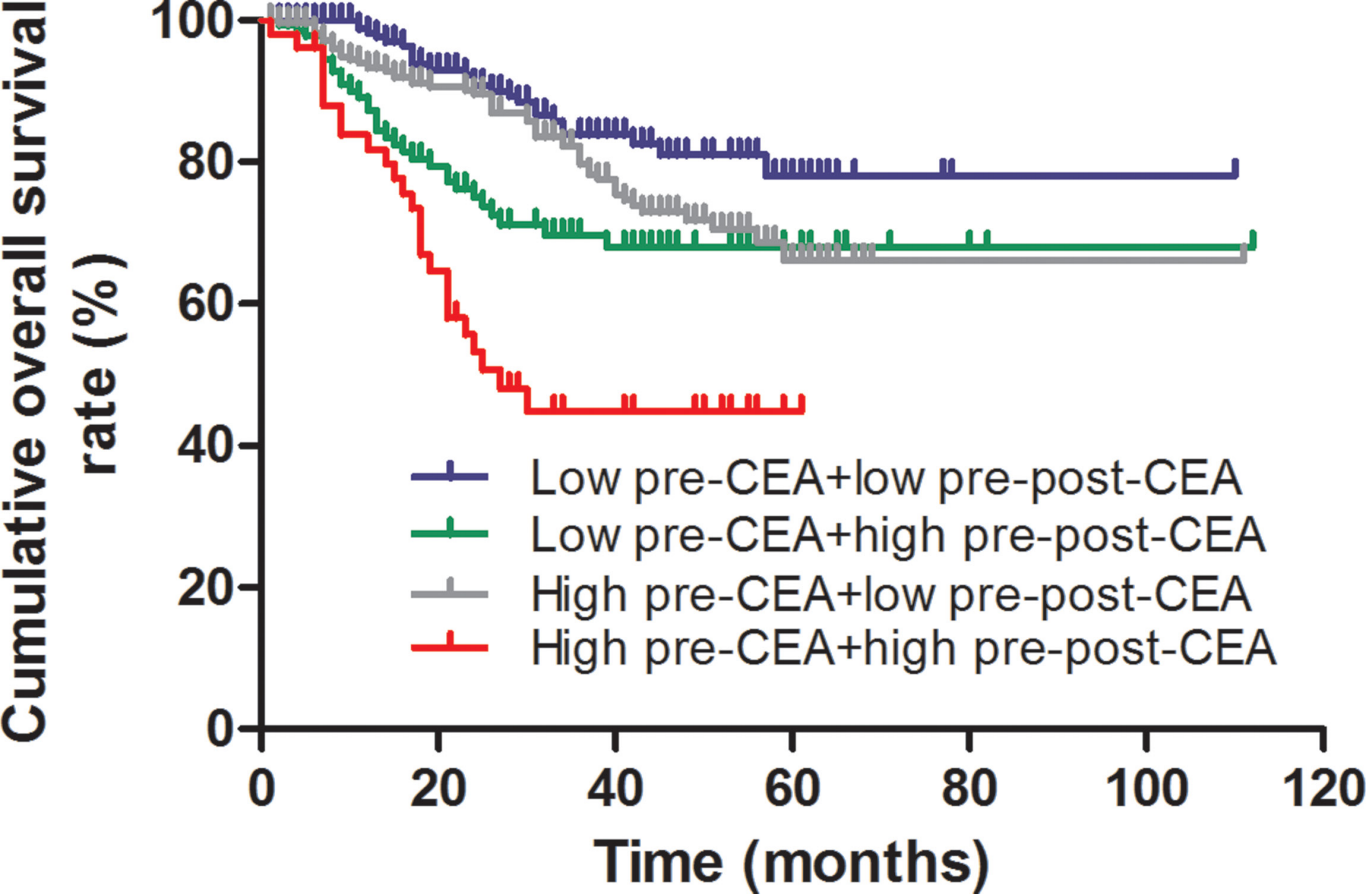
Kaplan-Meier survival curve analysis of different combinations of pre-CEA levels and pre-post-CEA ratios CRC patients with low pre-CEA levels plus low pre-post-CEA ratio (blue) survived longer than those with high pre-CEA levels plus high pre-post-CEA ratios (red). CRC patients with low pre-CEA levels plus high pre-post-CEA ratios (green) and those with high pre-CEA levels plus low pre-post-CEA ratios (gray) show comparable survival rates (log-rank test, *P* > 0.05) and are intermediate in comparison to the previous two groups (log-rank test, all *P* > 0.05).

Kaplan-Meier survival curve analysis showed that overall survival decreased post-operatively (Figure 2). Interestingly, patients with low pre-CEA levels demonstrated survived longer than those with high pre-CEA levels (log-rank test, *P* < 0.001; Figure 3). Meanwhile, patients with a lower pre-post-CEA ratios survived longer compared to those with higher pre-post-CEA ratios (log-rank test, *P* < 0.001; Figure 4). Also, patients with low pre-CEA levels and pre-post-CEA ratios (group 1) showed longer survival than patients with high pre-CEA levels and pre-post-CEA ratios (group 2). However, patients with lower pre-CEA levels, but high pre-post-CEA ratios (group 3) and patients with higher pre-CEA levels and low pre-post-CEA ratios (group 4) showed no differences with overall survival (log-rank test, *P* > 0.05). Also, patients in groups 3 and 4 showed lower OS than group 1 patients and higher OS than group 2 patients (log-rank test, both *P* > 0.05; Figure 5). This suggested that the pre-CEA levels and pre-post-CEA ratios had a synergistic effect in prognostic prediction of overall survival of CRC patients.

## DISCUSSION

Carcino-embryonic antigen (CEA) represents a group of macromolecular acid glycoproteins expressed highly during fetal development and reduced in the adults [14, 15]. High CEA levels were first observed in colorectal cancer and then in other malignant tumors of the esophagus, stomach, liver and pancreas [16, 17]. CEA is secreted by cancer cells resulting in high serum CEA levels.

Many studies have suggested that CEA levels can be used for predicting metastasis although few contradictory reports exist [18–20]. High CEA levels are observed in cancers, whereas changes are also observed in smoking, inflammation, pregnancy, gynecological disease and hepatitis conditions [21, 22]. Furthermore, prognostic potential of pre-post-CEA ratio has been reported for CRC patients. In gastric cancer, elevated pre-operative CK19 and CEA mRNA levels were associated with lymph node metastasis and predicted poor prognosis [22]. Yang *et al*. also reported that elevated pre- and post-operative serum CEA levels were associated with recurrence and poor clinical outcomes in few CRC patients [23]. Duan *et al* showed that high serum CEA or CYFRA 21-1 levels before and after surgery were associated with poor overall survival (OS) and progress-free survival (PFS) in stage I non-small cell lung cancer [11]. In this study, we explored the prognostic significance of pre-post-CEA ratios for CRC patients. We observed that pre-post-CEA ratios were closely related with smoking, tumor size, lymph node and distant metastases and post-operative pathological TNM staging. Also, our analysis demonstrated that distant metastasis and post-operative TNM stage were independent risk factors along with pre-CEA levels and pre-post-CEA ratios, consistent with previous reports.

Lymph node metastasis [24] and distant metastasis, particularly liver [25, 26] are independent risk factors with pre-CEA levels for CRC. The cell recognition, adhesion and immunosuppressive properties of CEA, which are Ca2+ dependent cell adhesion molecules aids aggregation, adhesion, invasion and metastasis of tumor cells [27, 28]. Therefore, excessive serum CEA levels are beneficial to CRC metastasis. Our study also demonstrated that lymphatic and distant metastases were independent risk factors in advanced stage III and IV CRC.

In previous studies, pre-CEA levels were identified as reliable indicators of prognosis and therapeutic efficacy in stage II and III CRC patients [18, 29]. Many studies also reported that pre-CEA levels were an independent risk factor for CRC patients [18, 30–31]. Our study further confirmed that pre-CEA level was an independent prognostic factor. Therefore, pre-CEA levels can be used evaluate therapeutic efficacy, post-operative tumor recurrence and predict prognosis of CRC patients.

Apart from the prognostic value of pre-CEA levels, few studies have also reported the prognostic value of pre-operative and post-operative CEA levels in combination [10]. Therefore, we analyzed the significance of pre-post-CEA ratios and found that it was associated with lymphatic and distant metastases and post-operative pathological TNM stage. These three indicators were also shown to be independent risk factors along with pre-post-CEA ratios.

Based on tumor source, the degree of malignancy determined by histology has been used to classify different pathological types of tumors [32, 33]. Most CRC patients are associated with moderately differentiated tumors, whereas high CEA levels are associated with poorly differentiated or undifferentiated CRC that are highly malignant [34]. In our study, pre-operative serum CEA levels were higher in lowly differentiated and undifferentiated carcinomas. Further, the differentiation degree of tumors affected the pre-post-CEA ratios in CRC patients. CEA is an important risk factor affecting CRC prognosis [13]. We observed that patients with decreasing post-operative CEA demonstrated better prognosis than those with higher post-operative CEA. Therefore, pre-post-CEA ratio is potentially an important prognostic marker.

During malignant transformation of CRC cells, overexpression of CEA lowers the function of other adhesion molecules and interferes in the interaction between normal cells, thereby weakening the single-layer structure of glandular tube [27–35, 36]. Therefore, excessive CEA in combination with genetic damage would promote malignancy of tumors. According to the NCCN guidelines, CRC of TNM stages 3 and 4 are accompanied by lymphatic and distant metastases, severely damaged colon tissues and CEA overexpression. Therefore, high pre-CEA levels and lymphatic or distant metastases were used as prognostic indicators and lymph node or micro-distant metastasis was neglected during surgery [37]. After surgery, post-CEA levels would increase leading to high pre-post-CEA ratios. In our study, pre-post-CEA ratio was closely related with lymphatic and distant metastases, TNM stage and degree of tumor differentiation, consistent with previous reports.

There is consensus that lymphatic metastasis, distant metastasis and TNM stage dictate prognostic status of CRC patients. In our study, these three indicators were also independent risk factors in relation to for pre-post-CEA ratios. Therefore, pre-post-CEA ratios are an essential risk factor for prognosis of CRC patients.

In conclusion, pre-post-CEA ratios were associated with the malignant phenotype of lymphatic metastasis, distant metastasis, TNM stage and degree of tumor differentiation in CRC patients. It was also an independent risk factor for CRC patients. Therefore, we postulate that pre-post-CEA ratio is a potential tumor biomarker to evaluate prognosis and therapeutic efficacy in CRC patients.

## MATERIALS AND METHODS

### Patients and clinicopathological parameters

This study was approved by the Ethics Committee of the First Affiliated Hospital of Zhengzhou University (No.ZZUF-2016174). All patients provided informed written consent prior to their participation. Clinicopathological parameters and follow-up data were obtained 2035 CRC patients (1138 male, 897 female) that received radical surgery in the First Affiliated Hospital of Zhengzhou University between June 2001 and June 2011. The mean patient age was 59.84+13.44 years. Among the 2035 CRC patients, 872 had colon cancer and 1163 rectal cancers. Colon cancer diagnosis was confirmed by histopathology prior to their participation. None of the enrolled patients received pre-operative chemotherapy or immunotherapy. Tumor-node-metastasis (TNM) stage was determined according to the American Joint Committee on Cancer/International Union Against Cancer TNM staging system of colorectal cancer (2010, 7th edition).

### Enzyme linked immunosorbent assay (ELISA) assay

Blood samples (4ml each) were obtained within the week before surgery for preoperative CEA estimation and 1–2 weeks after the surgery for post-operative CEA estimation. Serum CEA levels were estimated by ELISA kit (ab183365, Abcam, England) according to manufacturer's instructions.

### Patient follow-up

After surgery, the patients were assessed once a month for the first 6 months, once every 3 months for the first 2 years, once every 6 months until 5 years followed by once a year. Follow-ups were either by outpatient or inpatient review or by telephone. 216 patients did not participate in the follow-up analyses because they did not communicate with the physicians after surgery. In addition, 13 patients developed dysthymia and were unable to cooperate with the study; 3 patients committed suicide and 19 patients did not participate in the follow-up for unknown reasons. Therefore, the total follow-up rate in the study was 87.67%.

### Chemotherapy and radical surgery

FOLFOX6 was used as the first line adjuvant or neoadjuvant therapy scheme for CRC patients with high-risk IInd or IIIrd stage. CapeOX was used as the chemotherapy scheme as either 1st or 2nd line adjuvant or neoadjuvant therapy for CRC patients with high-risk IInd, IIIrd or IVth stage, drug resistance or postoperative recurrence. FOLFIRI was used as the chemotherapy scheme for CRC patients in advanced IVth stage, postoperative recurrence, metastasis or drug resistance.

Radical surgery was performed according to complete mesocolic excision (CME) for colon cancer patients and total mesorectal excision (TME) principle for rectal cancer patients.

### Statistical analysis

All statistical analyses were carried out using SPSS for Windows version 20 (SPSS Inc., Chicago, IL, United States). Univariate analysis was performed by χ^2^ test to analyze pre-CEA levels and pre-post-CEA ratios and clinicopathological parameters. Kaplan Meier survival curves and log-rank test were used to compare the low and high level groups of pre-CEA levels and pre-post-CEA ratios. Multivariate survival analysis was performed by the Cox regression model to determine relative risk (RR) and 95% confidence intervals (CI). Statistical significance was defined as *P* < 0.05.
